# Recent Advances in Exopolysaccharides from *Paenibacillus* spp.: Production, Isolation, Structure, and Bioactivities

**DOI:** 10.3390/md13041847

**Published:** 2015-04-01

**Authors:** Tzu-Wen Liang, San-Lang Wang

**Affiliations:** Department of Chemistry/Life Science Development Centre, Tamkang University, New Taipei City 25137, Taiwan; E-Mail: ltw27@ms55.hinet.net

**Keywords:** *Paenibacillus* spp., exopolysaccharides, squid pen, production, purification, bioactivity

## Abstract

This review provides a comprehensive summary of the most recent developments of various aspects (*i.e.*, production, purification, structure, and bioactivity) of the exopolysaccharides (EPSs) from *Paenibacillus* spp. For the production, in particular, squid pen waste was first utilized successfully to produce a high yield of inexpensive EPSs from *Paenibacillus* sp. TKU023 and *P. macerans* TKU029. In addition, this technology for EPS production is prevailing because it is more environmentally friendly. The *Paenibacillus* spp. EPSs reported from various references constitute a structurally diverse class of biological macromolecules with different applications in the broad fields of pharmacy, cosmetics and bioremediation. The EPS produced by *P. macerans* TKU029 can increase *in vivo* skin hydration and may be a new source of natural moisturizers with potential value in cosmetics. However, the relationships between the structures and activities of these EPSs in many studies are not well established. The contents and data in this review will serve as useful references for further investigation, production, structure and application of *Paenibacillus* spp. EPSs in various fields.

## 1. Introduction

Natural polysaccharides are highly susceptible to natural biodegradation and are less harmful than synthetic polymers. Over the past few decades, the number of known polysaccharides produced by microbial fermentation has been gradually increasing. Most of the polysaccharides derived from microorganisms are of the exo-polysaccharide (EPS) type; these EPSs attach to the cell surfaces or are released into the extracellular medium [1,2]. The microbial exo-polysaccharides can be divided into homo-polysaccharides, which are composed of a single monosaccharide unit, and hetero-polysaccharides, in which regular repeat units are formed from two to eight monosaccharides [3]. The repeating units of these EPSs are very regular, branched or unbranched, and interconnected by glycosidic linkages. Microbial EPSs are water-soluble polymers and may be ionic or non-ionic in nature. In addition, they are usually biodegradable, biocompatible, edible and nontoxic toward humans and the environment. Due to their many interesting physico-chemical and rheological properties in that they disperse in water to give a thickening or a viscosity building effect [4]. The shear thickening effect of EPSs is one in which viscosity increases with the rate of shear strain. Microbial EPSs are generally of uniform structure and fairly limited polydispersity, which depend on their molecular weights and structure. For example, the microbial levan similar to bacterial dextran is a polymer of fructose linked by β-(2→6) fructofuranosidic bond. It is known that levans with different molecular weights are needed for different purposes. The low molecular-weight, less branched levan usually provides a low viscosity, and can be used as a tablet binder in immediate-release dosage forms, while levans of medium- and high-viscosity grade are used in controlled-release matrix formulations [5]. Besides, curdlan, a linear glucan interconnected by β-(1→3) glucosidic linkages, is produced by the *Agrobacterium* species or *Alcaligenes faecalis* in initial studies [6]. This biopolymer is a thickener with unique thermal gelling properties; hence it is a good gelling material for the improvement of the textural quality, water-holding capacity and thermal stability of various commercialized products in food industry [6]. Microbial EPSs have found many applications in the food, pharmaceutical and other industries, such as the production of textiles, detergents, adhesives, cosmetics, microbial enhanced oil recovery, controlled drug delivery and wastewater treatment [6,7] because they showed properties of various physiological activities, stabilization, suspension of particulates, control of crystallization, inhibition of synaeresis, encapsulation and formation of film [8,9,10]. 

In recent years, significant progress has been made in the search for novel microbial EPSs that possess novel and highly functional properties [5]. The different biopolymers that have been extensively studied and are currently being marketed as commercial products include hyaluronic acid from *Streptococcus equii* and *Streptococcus zooepidemicus* [11]; levan from *Bacillus subtilis* and *Bacillus polymyxa* [12,13]; pullulan from *Aureobasidium pullulans* [14]; dextran from *Leuconostoc mesenteroides* [15]; xanthan from *Xanthomonas campestris* [16]; gellan from *Sphingomonas paucimobilis* [17]; alginate from *Pseudomonas* species and *Azotobacter chrococcum* [18]; cellulose from *Acetobacter xylinium* [19]; curdlan from *Agrobacterium* and *Alcaligenes* species [20]; and succinoglycan from *Rhizobium* [1]. However, the novel EPSs from *Paenibacillus* spp. were only discovered in the past ten years.

The genus *Paenibacillus* consists of over 89 species of facultative anaerobes, endospore-forming, neutrophilic, periflagellated heterotrophic and low G + C Gram-positive bacilli, which were originally included within the genus *Bacillus* and then reclassified as a separate genus in 1993 [21]. Bacteria belonging to this genus have been detected in a variety of environments, such as soil, water, rhizospheres, vegetable matter, forage and insect larvae, as well as clinical samples [7]. *Paenibacillus* spp. produced a wide variety of different EPSs with diverse physiological and biotechnological functions. *Paenibacillus* spp. EPSs have also attracted great interest because of their biotechnological potential in different industrial processes and wastewater treatment [6,7,22,23,24,25,26,27,28,29,30,31,32]. Thorough reviews on the production and applications of *Paenibacillus* spp., EPSs have shown that there is a growing interest in using *Paenibacillus* spp. EPSs as biomaterials; extensive research has been performed, leading to a large number of publications in recent years [6,7,22,23,24,25,26,27,28,29,30,31,32]. The present review is devoted to a survey of the main achievements in the production, isolation and structural composition of *Paenibacillus* spp. EPSs. Furthermore, the applications of *Paenibacillus* spp. EPSs in various fields are extensively reviewed.

## 2. Production of EPSs from *Paenibacillus* spp. 

As mentioned earlier, *Paenibacillus* spp. were discovered to produce various EPS. Some of the production conditions have been investigated to optimize the production for commercial usage. The production of EPS is not species-specific and each strain of the species produces different types of EPSs with different biotechnological properties. In our reviews, very recently, researchers observed that the culture of *Paenibacillus* spp. could produce two different, commercial, well-known EPSs. One of these polymers is a levan-type EPS from *P. polymyxa* EJS-3 [25], and the second is curdlan gum from *P. polymyxa* ATCC 21830 [6]. Levan is a polymer of fructose linked by a β-(2→6) fructofuranosidic bond and is present in many plants and microbial products [33]. The microbial levans are produced from sucrose-based substrate by a transfructosylation reaction of levansucrase (beta-2,6-fructan:d-glucose-fructosyl transferase, EC 2.4.1.10) by microorganisms. Liu *et al.* [23,24,25,29] reported that the shake flask cultures of the endophytic bacterium *P. polymyxa* EJS-3 grown in a culture medium (sucrose 188.2 g/L, yeast extract 25.8 g/L, K_2_HPO_4_ 5 g/L, CaCl_2_ 0.34 g/L, and initial pH 8.0) typically yielded 35.26 g/L of a levan-type EPS after cultivation for 60 h at 24 °C. Curdlan is a neutral, bacterial extracellular polysaccharide without branched chains, composed entirely of β-(1→3)-d-glycosidic linkages. Curdlan is a thickener with unique thermal gelling properties and is widely used in the food industry. Curdlan is biodegradable, nontoxic toward humans and the environment, edible and it has growing capacity in the pharmaceutical industry because of its potent biological activities [6]. Modified curdlan has pharmaceutical applications, such as controlled drug delivery [34].

To improve the efficiency and productivity of *Paenibacillus* spp. EPSs fermentation processes, many investigators have studied the effects of various process factors on the maximal production of EPSs to optimize the fermentation conditions, such as medium composition, temperature, pH, and culture vessel [6,7,22,23,24,25,26,27,28,29,30,31,32]. Table 1 provides a summary of the strains, culture conditions and EPS yields of *Paenibacillus* spp. that have been reported in the literature. The EPS yields varied over a wide range from 3.44 to 41.25 g/L with the bacterial species and culture conditions. In particular, our group previously demonstrated that EPSs were induced from a squid pen powder (SPP)-containing medium by *Paenibacillus* sp. TKU023 [31] and *P. macerans* TKU029 [32]. The production of inexpensive EPS is an important factor in the utilization of fishery waste products. The discovery of an inexpensive EPS not only solves environmental problems but promotes the economic value of marine waste. Until now, there have only been some reports on the culture conditions for the production of EPSs from *Paenibacillus* spp. Thus, there is a need to enhance the EPS productivity from *Paenibacillus* spp. through effective strategies of process intensification in the future.

### 2.1. Influence of Carbon/Nitrogen (C/N) Sources, pH and Temperature

Studies of EPS have indicated that the medium composition plays a critical role in EPS production [23,35]. The most commonly used carbon sources for EPS production are sugars, namely glucose and sucrose [6,23,27,28,29,30,36]. As shown in Table 1, sucrose is often employed as the most suitable carbon source for EPS production. It has been reported recently that the use of sucrose in the medium results in high yields of levan-type EPSs [23,29]. This is consistent with the findings of many other earlier investigators [33,37]. It has been reported that levansucrase with strong sucrose hydrolysing activity is involved in many *P. polymyxa* strains, which may be responsible for the high yield of EPS with sucrose as a carbon source [33,35,37]. However, the high cost of these carbon sources has a direct impact on production costs, which limits the market potential of these biopolymers. To decrease the production costs, it is important to look for less expensive carbon sources, such as wastes or industrial by-products [9]. Fishery by-products contain a large amount of chitin, which is a long-chain polysaccharide of a *N*-acetylglucosamine. Therefore, to lower the production cost of EPSs and efficiently reutilize the chitin-containing fishery by-products, we screened and isolated EPS-producing bacteria using squid pen powder (SPP) as the sole C/N source. We first produced and characterized the EPSs from the SPP culture supernatant of two bacteria, *Paenibacillus* sp. TKU023 [31] and *P. macerans* TKU029 [32]. *Paenibacillus* sp. TKU023 and *P. macerans* TKU029 have a chitin hydrolysing mechanism because there is EPS production by TKU023 and TKU029 with SPP as the C/N source without sucrose (Table 1). For media containing 1.5% (w/v) and 2% (w/v) SPP, the EPS yields from *Paenibacillus* sp. TKU023 and *P. macerans* TKU029 were 4.55 g/L and 3.46 g/L, respectively. However, in other literature, using high concentrations of sucrose in the medium resulted in EPS yields increasing up to 25.63 g/L [27] and 35.26 g/L [23,29]. This inspired us to increase the concentration of SPP to obtain higher yields of EPSs. The results demonstrated that when media contained 10% (w/v) SPP, the EPS yields from *Paenibacillus* sp. TKU023 and *P. macerans* TKU029 were 41.25 g/L and 35.75 g/L, respectively. Additionally, during fermentation, due to liquefaction of protein and chitin, bioactive material rich liquor is formed, including enzymes, biosurfactants, peptides, and chitooligosaccharides [31,32,38]. Furthermore, the fermented SPP in the culture broth could be recovered for biological applications in dye removal [39]. These advantages of using SPP as the sole C/N source to produce EPS are different from using sucrose.

**Table 1:** EPS production by fermentation of *Paenibacillus* spp.

Bacteria Source	Fermentation Conditions					EPS Yield (g/L)	References
	Medium Composition	Temperature (°C)	pH	Culture Vessel	Period (Days)		
*Paenibacillus* sp. TKU023	1.5% SPP, 0.1% K_2_HPO_4_, and 0.05% MgSO_4_·7H_2_O	37	7.23	50 mL in a 250 mL flask at 150 rpm	5	4.55	[31]
	10% SPP, 0.1% K_2_HPO_4_, and 0.05% MgSO_4_·7H_2_O	37	7.23	50 mL in a 250 mL flask at 150 rpm	5	41.25	
*P. macerans* TKU029	2% SPP, 0.1% K_2_HPO_4_, and 0.05% MgSO_4_·7H_2_O	30	7.21	100 mL in a 250 mL flask at 150 rpm	4	3.46	[32]
	10% SPP, 0.1% K_2_HPO_4_, and 0.05% MgSO_4_·7H_2_O	30	7.21	100 mL in a 250 mL flask at 150 rpm	4	35.75	
*P. polymyxa* SQR-21	Galactose 48.5 g/L, Fe^3+^ 242 µM and Ca^2+^ 441 µM	30	6.5	250 mL in a 1 L flask	4	3.44	[7]
*P. polymyxa* DSM 365	5 g/L yeast extract	30–40		1 L in 2 L Jar fermenter: agitation speed, 500 rpm			[22]
*P. polymyxa* EJS-3	Sucrose 188.2 g/L, yeast extract 25.8 g/L, K_2_HPO_4_ 5 g/L, CaCl_2_ 0.34 g/L	24	8	200 mL in a 1 L flask	2.5	35.26	[23,24,25,29]
*P. jamilae* CP-38	80% olive mill wastewaters (OMW)	30	7	2 L bioreactor at 150 rpm	3	4.2	[26]
*P. elgii* B69	Sucrose 51.35 g/L, peptone 6.78 g/L and yeast extract 0.47 g/L	30	7.2	100 mL in a 250 mL flask at 220 rpm	4	25.63	[27]
*P. polymyxa* ATCC 21830	Glucose 100 g/L, yeast extract 3 g/L	50	7	400 mL in a 1 L flask at 150 rpm	4	6.89	[6]
*P. polymyxa*	Sucrose 20 g/L, yeast extract 0.2 g/L, K_2_HPO_4_ 0.25 g/L, MgSO_4_ ·7H_2_O 0.1 g/L, NaCl 0.05 g/L, agar 15 g/L	30		Petri dishes of 90 mm of diameter	5		[28]
*P. polymyxa* JB115	MSM broth containing 10% sucrose	30		1 L medium at 180 rpm	3	10	[30]

Many results have also reported that the production of EPS is cell growth-associated [7,31,32]. In addition to the C/N source, the initial liquid culture pH and culture temperature are both important and may affect the cell growth, the uptake of different nutrients and EPS production. An optimal value of pH for the EPS production from *Paenibacillus* spp. lies in the range between 6.5 and 7.2 (Table 1) [6,7,22,26,27,31,32]. Rafigh *et al.* found that when the initial pH of the fermentation broth was increased from 5.5 to 7.0, there was an increase in curdlan gum and biomass production, approximately 39.3% and 4.8%, respectively. However, higher values (pH 8.5) caused a decrease in their production [6]. These EPSs from *Paenibacillus* spp. were in agreement with previous study that for EPS production by *P. polymyxa* KCTC 8648P, the optimum pH value of 7.0 was reported [37]. However, there were only a few reports where the highest EPS content was achieved by cultivating the microorganism at slightly alkaline pH [23,24,25,29]. In the experiments with the strain *P. polymyxa* EJS-3, Liu *et al.* [23,24,25,29] found that the specific growth conditions of *P. polymyxa* EJS-3 might be due to its living in the tissues of plants.

Incubation temperature is another critical factor for EPS biosynthesis [23]. Rafigh *et al.* [6] found that when the temperature varied from 30 to 40 °C, the yield of curdlan production increased rapidly and then slightly increased as the fermentation proceeded from 40 to 50 °C. Batch fermentation processes were conducted at 25 °C separately, but the yield of curdlan production was very low, only a few µg/L. In addition, above 50 °C, curdlan may be curdled. Therefore, a higher temperature ratio was not employed in the production of curdlan [6]. However, Liu *et al.* reported that the optimal temperatures of *P. polymyxa* EJS-3 for cell growth and EPS production were 27 °C and 24 °C, respectively [23]. *P. polymyxa* EJS-3 favoured lower temperature for EPS production compared with others.

### 2.2. Fermentation Techniques

The influence of aeration on the vital activity of *Paenibacillus* spp. producing EPS was studied [6,31,32]. Under anaerobic conditions, the cell population neither grows nor produces EPS. An intense aeration during fermentation leads to a significant increase of the EPS concentration. Previous studies also argued that agitation could be beneficial to the growth and performance of the microbial cells through improving the characteristics of mass transfer with respect to substrates, products and oxygen [40]. The findings by Rafigh *et al.* [6] also indicated that both biomass and curdlan production from *P. polymyxa* ATCC 21830 underwent significant enhancements as the agitation speed increased from 120 to 150 rpm. The highest yield of curdlan gum and biomass production was at an agitation speed of 150 rpm. The curdlan gum and biomass production were low at 120 rpm, which can be attributed to the limitation of oxygen transfer. However, at 180 rpm, lower levels of curdlan gum and biomass were observed, which can be ascribed to bacterial fragmentation mediated by several shearing mechanisms. However, for some other species of strains, higher agitation speed (600 rpm) has been used for curdlan production, e.g., from *Agrobacterium* sp. [20], but not from *Paenibacillus* sp.

## 3. Isolation and Purification of EPS

**Figure 1:**
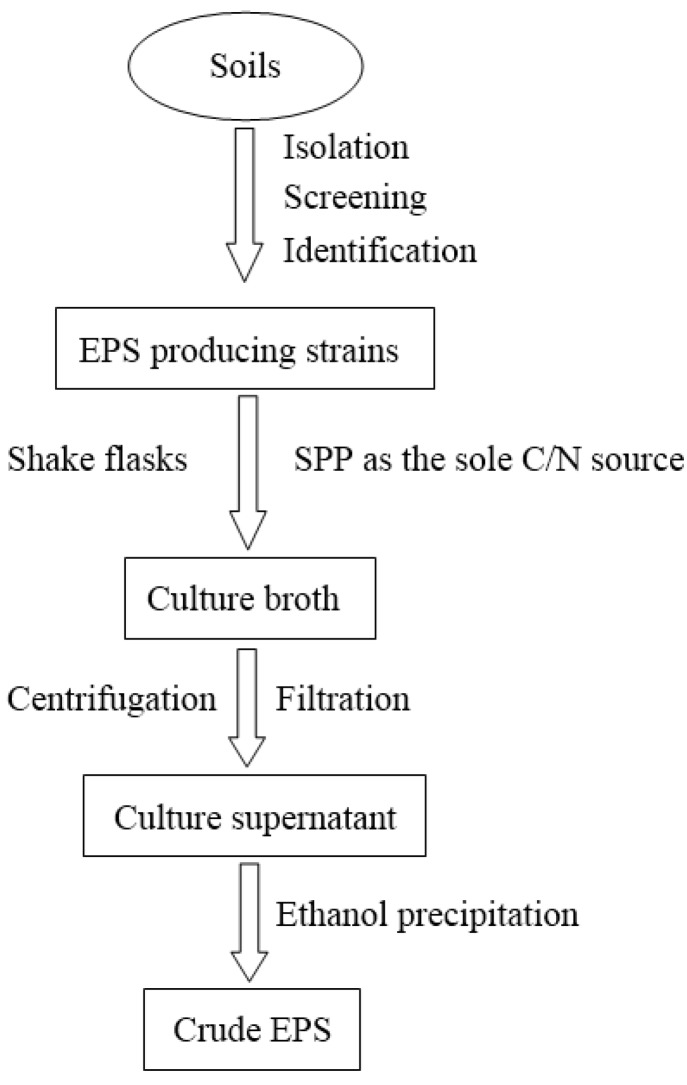
Schematic diagram for the isolation of EPS from *Paenibacillus* spp.

In our previous studies, the EPS sample after SPP fermentation was immediately autoclaved for 20 min to reduce the ropy condition of the culture and was centrifuged (12,000 *g* for 20 min) to remove the remaining SPP and biomass. The supernatant was filtered through a 0.45-μm membrane filter, mixed with two volumes of methanol, stirred vigorously and kept overnight at 4 °C. The precipitate from the ethanol dispersion was collected by centrifugation at 12,000 *g* for 15 min, washed three times with sterilized distilled water, and then lyophilized to yield the crude EPS. This is the most common and convenient method for isolating EPS from culture supernatant [7,23,27,30,31,32]. Figure 1 summarizes the isolation procedures of EPS from *Paenibacillus* spp.

The crude EPS was re-dissolved in distilled water and stirred vigorously at 80 °C for 30 min, mixed with four volumes of anhydrous ethanol, stirred vigorously and kept overnight at 4 °C. The precipitate from the ethanol dispersion was collected by centrifugation at 12,000× *g* for 15 min, re-dissolved in distilled water and deproteinized with 1/5 volume of Sevag reagent (CHCl_3_-BuOH, v/v = 5/1) seven times [41]. The deproteinized solution was then dialyzed against distilled water, concentrated and lyophilized to yield deproteinized EPS.

The deproteinized EPS was purified sequentially through column chromatography, including ion-exchange chromatography, gel filtration chromatography and affinity chromatography [7,23,27,30,31,32]. Elution was conducted with an appropriate running buffer, followed by collection, concentration, dialysis, and lyophilization, and the carbohydrates were determined by the phenol-sulfuric acid method using glucose as the standard [42]. In addition, based on the different solubility of the EPSs in ethanol, isopropanol, and other solvents, the EPSs were simply and effectively fractionated. Huang *et al.* recently isolated EPS fractions from a fermentation medium by gradient ethanol precipitation [43]. Their results suggest that the method is simple and workable for the initial fractionation of EPSs, proteins, and their complexes with different molecular sizes and for further identification of bioactive components.

## 4. Physicochemical Characterization

*Paenibacillus* spp. EPSs with different monosaccharide constituents and chemical structures have been isolated from their culture supernatant. Many research groups have elucidated the chemical structures of purified EPSs using infrared spectroscopy, liquid-state nuclear magnetic resonance (NMR) (one and two dimensions), solid-state NMR, gas chromatography (GC), GC-mass spectroscopy (GC-MS), high-performance liquid chromatography (HPLC), acid hydrolysis, methylation analysis, periodate-oxidation, and Smith degradation [7,23,27,30,44]. The different structural characteristics of EPS from *Paenibacillus* spp. showing various bioactivities have been investigated. The sources and chemical compositions of the EPSs are summarized in Table 2.

### 4.1. Molecular-Weight Distribution of EPS from Paenibacillus spp.

Various techniques, such as viscometry, osmometry, sedimentation, and HPLC, have been used to determine the average polymer molecular weight (MW) and polydispersity index. Among them, high-performance gel permeation chromatography (HPGPC) is a common method for determining the MW of polysaccharides and has also been used by many researchers for the MW of EPSs. Size-exclusion chromatography with multi-angle laser light scatter detection is also an efficient method for the evaluation of the absolute MW of polysaccharides and provides greater resolution than traditional GPC [6,7,23,27,30,45,46]. The average molecular weight of the EPS from *Paenibacillus* spp. varies in very broad ranges, from hundreds to thousands of kDa, depending on the culture strain, pH, cultivation techniques and C/N sources used. It has been reported that on a sucrose-containing medium, the bacterium produces high-molecular-weight EPS [23]. The molecular weights of EPS-1 and EPS-2 from *P. polymyxa* EJS-3 were estimated to be 1220 and 869 kDa, respectively [23]. The average molecular weight of curdlan produced using glucose as the carbon source by *P. polymyxa* ATCC 21830 was 170 kDa [6]. The molecular weight of EPS from *Paenibacillus* spp. varies significantly and is affected by variables such as temperature, pH and the initial carbon source concentration [11]. 

### 4.2. Monosaccharide Composition

The monosaccharide composition analysis usually involves cleavage of glycosidic linkages by acid hydrolysis, derivatization, and detection and quantification by GC. In addition, high-performance anion-exchange chromatography with pulsed amperometric detection has been developed to supplement traditional methods because it does not require derivatization of the monosaccharide with high resolution [47]. Recently, a 1-phenyl-3-methy-5-pyrazolone pre-column derivatization method has been used to determine the monosaccharide composition [48].

Many different EPSs from *Paenibacillus* spp. have been obtained; the monosaccharide composition is usually glucose, mannose, galactose and glucuronic acid in various mole ratios (Table 2). There is a large variety of EPS produced by *Paenibacillus* spp. depending on the type of *Paenibacillus* sp. strain, culture conditions and medium composition. The characterization of the EPS from *Paenibacillus* sp. TKU023 demonstrated that it was mainly composed of glucose and maltose [31]. Madden *et al.* reported that EPS produced by *P. polymyxa* NCIB 11429 was composed of glucose, mannose, galactose, glucuronic acid and pyruvate [49]. Han and Clarke found that EPS from *P. polymyxa* NRRL B-18475 was β-(2-6) linked fructan [33]. The EPS produced by *P. polymyxa* KCTC 8648P was composed of glucose, galactose, mannose, fucose and glucuronic acid [37]. Liu *et al.* reported that mannose, fructose and glucose were the constituents of the EPS from *P. polymyxa* EJS-3 [23]. The EPS from *P. polymyxa* SQR-21 was composed of mannose, glucose, fructose and glucuronic acid [7]. In particular, Li *et al.* found that the EPS produced by *P. elgii* B69 was composed of glucose, glucuronic acid, xylose, and mannose [27]. This finding differed from the EPS produced by other *Paenibacillus* strains. Xylose is rarely described as a main component of other bacterial polysaccharides. These dissimilarities reflect the species-specific production and biotechnological potential of EPS. Increasing numbers of microbial strains have been screened to produce bioactive EPS to replace synthetic chemicals, which have many side effects.

**Table 2:** Chemical structures of EPSs from *Paenibacillus* spp.

Microorganisms	Chemical Composition of EPSs	References
*Paenibacillus* sp. TKU023	glucose and maltose	[31]
*P. polymyxa* KCTC 8648P	Glucose, galactose, mannose, fucose and glucuronic acid	[37]
*P. polymyxa* NCIB 11429	Glucose, mannose, galactose, glucuronic acid and pyruvate	[49]
*P. polymyxa* NRRL B-18475	β-(2-6) linked fructan	[33]
*P. elgii* B69	Glucose:glucuronic acid:xylose:mannose = 1:0.53:1.15:0.46	[27]
*P. polymyxa* ATCC 21830	linear glucan interconnected by β-(1→3) glucosidic linkages	[6]
*P. polymyxa* JB115	glucan having β-(1,3) and β-(1,6) linkages	[30]
*P. polymyxa* EJS-3	Mannose, fructose and glucose	[23]
*P. polymyxa* SQR-21	Mannose, glucose, fructose and glucuronic acid	[7]

## 5. Bioactivities and Application of *Paenibacillus* spp. EPSs

In recent years, microbial EPSs and their derivatives have found many applications in the food, pharmaceutical and other industries because they have different physiological activities from natural gums and synthetic polymers [9,10]. Moreover, they are highly susceptible to natural biodegradation and are less harmful than synthetic polymers. Their applications are diverse, ranging from the laboratory through clinical to tableting; they have found applications in such diverse bio-medical fields as ophthalmology, orthopedic surgery, tissue engineering, implantation of medical devices and artificial organs, prostheses, dentistry, bone repair and many other medical fields [5]. In addition, they have therapeutic and pharmaceutical uses in that they enable the controlled, slow-release of drugs into the body. They also make possible targeting of drugs into sites of inflammation or tumors for disease treatment, and they can be used for skin rejuvenation and wound healing [5,32]. Several of these microbial polysaccharides are commercial industrial products, whereas others are in various stages of developments. The multiple bioactivities and environmental benefits of EPSs from *Paenibacillus* spp. are summarized in Table 3 and are compared in detail below.

**Table 3:** Potential applications of EPSs from *Paenibacillus* spp.

Microorganisms	EPSs	Applications	References
*Paenibacillus* sp. TKU023		Antioxidant	[31]
*P. macerans* TKU029		Improvement of human skin hydration	[32]
*P. polymyxa* EJS-3	Levan and its derivatives	Antioxidant Antitumor	[23,24,25]
*P. jamilae* CP-38		Reduction in the toxicity of olive mill wastewaters Heavy metal biosorption capacity	[26]
*P. elgii* B69		Bioflocculant	[27]
*P. polymyxa* ATCC 21830	Curdlan	Drug-delivery carriers for the sustained release of drugs and a support matrix for immobilization of enzymes	[6]
*P. polymyxa*		Removal of cadmium	[28]
*P. polymyxa* SQR-21		Antioxidant Bioflocculant Metal chelating capacity	[7]
*P. polymyxa* JB115	Glucan	Animal feed additive for the purpose of enhancing immunity	[30]

### 5.1. Antioxidant and Antitumor Activity

Free radicals are harmful to living organisms [50]. To reduce the damage caused by free radicals, both synthetic and natural antioxidants are used. However, synthetic antioxidants are thought to cause liver damage and carcinogenesis [23]. Therefore, it is essential to develop natural nontoxic antioxidants to protect humans from free radicals. Novel natural antioxidants have gained importance in science and medicine in recent decades. The antioxidant properties of EPSs have been reported from many types of EPSs derived from filamentous fungi [51], such as *Cordyceps militaris* SU5-08 [51], *Fusarium solani* SD5 [50], *Pleurotus sajor-caju* [52], *Fomes fomentarius* [53], *Tremella fuciformis* [54], *Agrocybe cylindracea* [55], *Collybia maculate* [56], *Cordyceps jiangxiensis* [57] and *Tremella mesenterica* [58]. However, relative to fungal EPS, reports concerning the antioxidant activities of bacterial polysaccharides [59,60,61] are more rare, such as *Paenibacillus* spp. EPSs. 

Antioxidant activities have been attributed to various reactions and mechanisms. The *in vitro* antioxidant capacities of EPS were evaluated using various assay methods and activity indices [7,23,24,25,29,31]. In our previous studies, *Paenibacillus* sp. TKU023 could produce EPS and antioxidant by using SPP as the sole C/N source. The culture supernatant incubated for four days using a baffled base flask showed strong 2,2-diphenyl-1-picrylhydrazyl (DPPH) radical scavenging activity, reducing powers, ferrous ion chelating activity and high total phenolic content, but maximum EPS production was found at the fifth day when using a flat base flask. The production of two invaluable environmentally friendly biomaterials (EPS and antioxidant) is unprecedented. In addition, the use of SPP (waste) is green, which made the whole process more valuable and attractive [31]. Raza *et al.* reported that *P. polymyxa* SQR-21 produced one type of EPS using yeast extract and galactose as the best N and C sources, respectively. Their EPS showed good superoxide scavenging and moderate inhibition of lipid peroxidation and reducing activities [7]. Furthermore, Liu *et al.* studied the *in vitro* and *in vivo* antioxidant activity of the levan-type EPSs from endophytic bacterium *P. polymyxa* EJS-3. In antioxidant assays *in vitro*, both crude EPS and its purified fractions (EPS-1 and EPS-2) were found to have moderate DPPH radical scavenging activity, hydrogen peroxide scavenging activity, lipid peroxidation inhibition effects, and strong ferrous ion chelating activity. In antioxidant assays *in vivo*, mice were subcutaneously injected with d-galactose for six weeks and administered EPS-1 via gavage. As a result, administration of EPS-1 significantly increased the thymus and spleen indices of d-galactose in aging mice. Moreover, EPS-1 administration significantly enhanced the activities of antioxidant enzymes and the total antioxidant capacity and decreased the levels of malondialdehyde in both serums and livers of aging mice [23,24]. Very recently, Liu *et al.* successfully acetylated, phosphorylated and benzylated the levan-type EPSs from *P. polymyxa* EJS-3 to obtain the derivatives of acetylated levan (AL), phosphorylated levan (PL) and benzylated levan (BL). For the antioxidant and antitumor activities *in vitro* of the natural polysaccharide and its derivatives, AL, BL and PL all exhibited higher reducing power, scavenging activity against superoxide radicals and scavenging activity of hydroxyl radicals compared to the natural polysaccharide, EPS-1. In addition, AL, BL and PL also exhibited higher antiproliferative activity against human gastric cancer BGC-823 cells *in vitro* than EPS-1. The enhanced activities of the derivatives were probably due to the introduction of acetyl, benzyl, or phosphoryl groups into the EPS-1 molecules, which increased the electron-donating ability and affinity with the receptors on immune cells. The results suggested that the derivatives could be explored as promising antioxidant and antitumor agents [25].

### 5.2. Improvement of Skin Hydration

Polysaccharides of bacterial origin are very important in the cosmetic and pharmaceutical industries [62]. One important criterion for the evaluation of cosmetic products is their effect on skin hydration. We found that EPSs from *P. macerans* TKU029 can significantly increase skin hydration [32]. The same amount of TKU029 EPSs (5%, w/v), propylene glycol/butylene glycol/water (1.5:1.5:1; v/v), and hyaluronic acid were applied to the skin of fifteen female volunteers (average age of 21 years). Skin hydration was measured in the test and control areas of each volunteer at each time point. The changes in skin capacitance 180 min after the application of TKU029 EPSs and hyaluronic acid were measured. TKU029 EPSs increased skin hydration significantly more than hyaluronic acid. During the application period, TKU029 EPSs increased skin hydration from 37.3% to 44.3%. Application of hyaluronic acid led to a slight increase at the beginning of the period, but then skin hydration decreased from 41.0% (at 30 min) to 37.0% (at 180 min). The untreated control remained nearly unchanged, *i.e.*, 37.3% at 0 min and 36.2% at 180 min. The untreated control revealed an increase in skin hydration after 180 min of 8.1% for TKU029 EPSs and of approximately 0.8% for hyaluronic acid [32]. The EPSs produced by *P. macerans* TKU029 can increase *in vivo* skin hydration and may be a new source of natural moisturizers with potential value in cosmetics.

### 5.3. Bioremediation of Wastewater

Chemical contamination of water from a wide range of toxic compounds, particularly dyeing pigments (from the textile industry), heavy metal ions, and other toxic suspended particles, remains a serious environmental problem causing serious risk to public health. Various physical and chemical processes have been developed for removing pollutants from wastewater. One of the most popular methods is flocculation due to its economic advantages and potency. Bioflocculants are more eco-friendly and biodegradable and are less harmful to the environment than inorganic flocculants and organic synthetic flocculants [63]. Many research groups have evaluated the flocculating activity of EPSs from *Paenibacillus* spp. Bioflocculation of high-ash Indian coals using *P. polymyxa* showed a 60% decrease in ash, suggesting that selective flocculation of coal is possible [64]. *P. polymyxa* P13 was reported as an EPS producer that exhibited significant biosorption capacity of Cu^2+^ produced in several industries [65]. The EPS from *P. polymyxa* SQR-21 showed a high flocculating activity towards activated carbon [7]. *P. jamilae* CP-38 was able to grow and produce EPS using olive mill wastewaters as the sole nutrient and energy source, with a concomitant reduction in the toxicity of the waste [26]. The EPS produced by *P. polymyxa* had cadmium sorption capacity in aqueous solution [28]. In particular, Li *et al.* discovered a new EPS-based broad-spectrum bioflocculant produced by a newly isolated strain, *P. elgii* B69 [27]. This bioflocculant had high activities towards all tested pollutants, including kaolin clay, dyeing pigment, heavy metal ion, and real wastewater. The multiple-pollutant-removal performance of *P. elgii* B69 is a significant advantage [27].

### 5.4. Other Bioactivities

As aforementioned, *Paenibacillus* spp. could produce different types of EPSs with different biotechnological properties. Many other important bioactivities of *Paenibacillus* spp. EPSs have also been investigated. *P. polymyxa* JB115 was isolated from Korean soil as a glucan producer for the development of animal feed additives showing activities as a biological response modifier, natural immuno-modulator and a potential anti-tumor agent for livestock [30]. Rafigh *et al.* demonstrated that *P. polymyxa* ATCC 21830 is capable of producing curdlan gum [6]. Curdlan has potential applications in the manufacture of food products, and it is also known as a drug-delivery carrier for the sustained release of drugs and as a support matrix for the immobilization of enzymes [66,67,68]. In addition, curdlan has been used together with activated carbon adsorbents for heavy metal removal [69]. 

## 6. Conclusions and Perspectives 

This review contains the most recent information on the production of various *Paenibacillus* spp. EPSs with applications in bioactivity and bioremediation. Economical, environmentally friendly and high quantity production of *Paenibacillus* spp. EPSs is necessary for various applications. Squid pen waste was utilized to produce high yield, inexpensive EPSs. The discovery of inexpensive EPSs not only solves environmental problems but also promotes the economic value of marine wastes. Furthermore, this EPS produced by *P. macerans* TKU029 using SPP can increase *in vivo* skin hydration and may be a new source of natural moisturizers with potential value in cosmetics. Novel uses of *Paenibacillus* spp. EPSs due to its inexpensive production and its bulk will be developed. In addition, the movement toward “greener” products and technologies that are more environmentally friendly is prevailing. Production methods of EPSs are eagerly anticipated in the future, and many have already reached the market. However, the relationship between the structural features, solution behavior, space conformation, and the bioactivity of *Paenibacillus* spp. EPSs is unclear due to the structural diversity and complexity of polysaccharide molecules. In addition, alteration of the chemical properties of the original EPSs will also greatly enhance their values and extend their range of applications. The elaboration of either biotechnological or technical procedures for the production of EPSs of diverse structures (e.g., varied stereochemical composition or molecular sizes) and ultimate product functions (e.g., varied water-solubility and physical activity) to meet the special demands of practical application are being launched and will soon provide a broad spectrum of new EPSs. These will be useful to understand the chemical structures, chain conformations and the biological activities for applications in various fields.
